# First detection and genotyping of *Enterocytozoon bieneusi* in pet fancy rats (*Rattus norvegicus*) and guinea pigs (*Cavia porcellus*) in China

**DOI:** 10.1051/parasite/2020019

**Published:** 2020-04-06

**Authors:** Jingsong Wang, Chaochao Lv, Diandian Zhao, Runan Zhu, Chen Li, Weifeng Qian

**Affiliations:** College of Animal Science and Technology, Henan University of Science and Technology Luoyang 471003 China

**Keywords:** *Enterocytozoon bieneusi*, Pet rats, Pet guinea pigs, Genotype, Zoonotic, China

## Abstract

*Enterocytozoon bieneusi*, an obligate intracellular microsporidian parasite, can infect humans and a wide variety of animals worldwide. However, information on the prevalence and molecular characterization of *E. bieneusi* in pet rats and guinea pigs is lacking. In this study, 325 fecal samples were collected from 152 pet fancy rats and 173 pet guinea pigs purchased from pet shops in Henan and Shandong provinces. The prevalence of *E. bieneusi* was 11.2% (17/152) in pet fancy rats and 20.2% (35/173) in pet guinea pigs. Genotypes D (*n* = 12), Peru11 (*n* = 3), S7 (*n* = 1) and SCC-2 (*n* = 1) were identified in pet fancy rats, and genotype S7 (*n* = 30) and a novel genotype PGP (*n* = 5) were identified in pet guinea pigs. The ITS sequence and its phylogenetic analysis showed that the novel genotype PGP was distinctly different; it exhibited less than 50% similarity to the reference sequences, and did not cluster with any of the known *E. bieneusi* genotype groups, forming a unique branch between groups 6 and 7. These data suggest that this is a new *E. bieneusi* genotype group. This is the first report of *E. bieneusi* infection in pet fancy rats and pet guinea pigs worldwide. The identification of zoonotic genotypes D, Peru11, and S7 suggests that pet fancy rats and guinea pigs can be potential sources of human microsporidiosis.

## Abstract (fr)

*Enterocytozoon bieneusi*, un parasite microsporidien intracellulaire obligatoire, peut infecter les humains et une grande variété d’animaux dans le monde. Cependant, les informations sur la prévalence et la caractérisation moléculaire d’*E. bieneusi* chez les rats et les cobayes de compagnie manquaient. Dans cette étude, 325 échantillons de matières fécales ont été prélevés de 152 rats et 173 cobayes achetés dans des animaleries dans les provinces du Henan et du Shandong. La prévalence d’*E. bieneusi* était de 11,2 % (17/152) chez les rats et de 20,2 % (35/173) chez les cobayes. Les génotypes D (*n* = 12), Peru11 (*n* = 3), S7 (*n* = 1) et SCC-2 (*n* = 1) ont été identifiés chez des rats de compagnie, et le génotype S7 (*n* = 30) et un nouveau génotype PGP (*n* = 5) ont été identifiés chez des cobayes de compagnie. La séquence d’ITS et son analyse phylogénétique ont montré que le nouveau génotype PGP était nettement différent ; la séquence présentait moins de 50 % de similitude avec les séquences de référence et ne se regroupait avec aucun des groupes de génotypes connus d’*E. bieneusi*, formant une branche unique entre les groupes 6 et 7 ; ces données suggèrent qu’il s’agit d’un nouveau groupe de génotype d’*E. bieneusi*. Ceci est le premier signalement d’infection par *E. bieneusi* chez des rats et des cobayes de compagnie dans le monde. L’identification des génotypes zoonotiques D, Peru11 et S7 suggère que les rats et les cobayes de compagnie peuvent être des sources potentielles de microsporidiose humaine.

## Introduction

*Enterocytozoon bieneusi*, a unicellular and obligate intracellular pathogen, has an extensive host range and has been identified in humans, livestock, companion animals, and wildlife, as well as in wastewater [17, 23]. *Enterocytozoon bieneusi* infection can cause self-limiting diarrhea, malabsorption, and wasting in immunocompetent hosts and life-threatening diarrhea in immunocompromized individuals [10]. Humans and animals can acquire infection via fecal–oral transmission of spores from infected individuals through direct contact or by consumption of contaminated food or water [17].

Genotyping based on the internal transcribed spacer (ITS) region of the rRNA gene has identified 11 major phylogenetic groups and more than 470 genotypes of *E. bieneusi* from various hosts [17]. To date, more than 60 *E. bieneusi* genotypes have been identified in rodents worldwide [6, 8, 12, 17, 24, 26, 30, 34]. For rats (*Rattus* spp.), only three surveys have focused on the molecular characterization of *E. bieneusi* in wild rats in Iran and China, and six genotypes (D, M, Peru6, CD6, BEB6, and CHG2) have been identified [24, 30, 34]. Only one published article has reported genotype peru16 from household guinea pigs in Peru [3].

Fancy rats, *Rattus norvegicus* forma domestica, are rodents belonging to the order Rodentia and family Muridae. Fancy rats have been bred as pets at least since the late 19th century; they are considered to be intelligent, playful, and trainable animals (http://www.afrma.org/). In recent years, fancy rats have become a very popular pet in China. Pet rodents can be hosts to several zoonotic pathogens, including viruses, bacteria, and parasites [20]; zoonotic transmission of *E. bieneusi* to a child from household guinea pigs has been reported [3]. However, no literature is available about the prevalence and genetic characteristics of *E. bieneusi* in pet rats and pet guinea pigs. Therefore, the aim of the present study was to determine the prevalence and genotypes of *E. bieneusi* in these animals and to assess its zoonotic potential.

## Materials and methods

### Ethics statement

The research protocol was reviewed and approved by the Research Ethics Committee of Henan University of Science and Technology.

### Sample collection

Between September 2018 and October 2019, 152 pet fancy rats and 173 pet guinea pigs were purchased from six pet shops in Luoyang, Henan and Weifang, Shandong, China (Tables 1 and 2). Upon arrival in the laboratory, each animal was immediately placed into a single clean plastic box for collection of fresh feces. A single sample was collected from each animal. All the specimens were refrigerated at 4 °C and DNA was extracted within one week. Only young pet fancy rats (4–10 week-old) and 1–8-month-old pet guinea pigs were available in these pet shops. All pet fancy rats and guinea pigs examined in this study were asymptomatic at the time of sample collection, and information on region, age, and sex of these animals was recorded.

**Table 1:** Prevalence and genotypes of *Enterocytozoon bieneusi* in pet fancy rats (*Rattus norvegicus*) in Henan and Shandong provinces, China.

Characteristics	No. of animals	No. positive (%)	Genotypes (no.)
Region			
Luoyang, Henan			
Pet shop 1	38	4 (10.5)	D (2), Peru11(2)
Pet shop 2	30	2 (6.7)	D (1), S7 (1)
Subtotal	68	6 (8.8)	D (3), Peru11(2), S7 (1)
Weifang, Shandong			
Pet shop 4	44	7 (15.9)	D (6), SCC-2 (1)
Pet shop 5	40	4 (10.0)	D (3), Peru11(1)
Subtotal	84	11 (13.1)	D (9), Peru11(1), SCC-2 (1)
Total	152	17 (11.2)	D (12), Peru11(3), S7 (1), SCC-2 (1)
Age (weeks)			
4–6	105	13 (12.4)	D (9), Peru11(2), S7 (1), SCC-2 (1)
7–10	47	4 (8.5)	D (3), Peru11(1)
Sex			
Male	85	11 (12.9)	D (8), Peru11(2), S7 (1)
Female	67	6 (9.0)	D (4), Peru11(1), SCC-2 (1)

**Table 2:** Prevalence and genotypes of *Enterocytozoon bieneusi* in pet guinea pigs (*Cavia porcellus*) in Henan and Shandong provinces, China.

Characteristics	No. of animals	No. positive (%)	Genotypes (no.)
Region			
Luoyang, Henan			
Pet shop 1	35	5 (14.3)	S7 (5)
Pet shop 2	32	7 (21.9)	S7 (5), PGP (2)
Pet shop 3	35	4 (11.4)	S7 (4)
Subtotal	102	16 (15.7)	S7 (14), PGP (2)
Weifang, Shandong			
Pet shop 5	35	6 (17.1)	S7 (6)
Pet shop 6	36	13 (36.1)	S7 (10), PGP (3)
Subtotal	71	19 (26.8)	S7 (16), PGP (3)
Total	173	35 (20.2)	S7 (30), PGP (5)
Age (months)			
1–3	122	29 (23.8)	S7 (26), PGP (3)
4–8	51	6 (11.8)	S7 (4), PGP (2)
Sex			
Male	76	16 (21.1)	S7 (14), PGP (2)
Female	97	19 (19.6)	S7 (16), PGP (3)

### DNA extraction

Each specimen was washed with distilled water by centrifugation for 10 min at 3000 ×*g* at room temperature. Before DNA extraction, 200 mg of each fecal sample was added to a 2 mL microcentrifuge tube containing 200 mg of glass beads, and were vortexed at maximum speed until the fecal samples were completely homogenized. Genomic DNA was extracted using an E.Z.N.A. Stool DNA Kit (Omega Bio-tek Inc., Norcross, GA, USA), according to the manufacturer’s instructions. The extracted DNA was kept at −20 °C before being used in PCR analysis.

### PCR amplification

*Enterocytozoon bieneusi* was examined by nested PCR targeting a ~390-bp fragment of the ITS region, as previously described [2]. The primers were EBITS3 (5′–GGTCATAGGGATGAAGAG–3′) and EBITS4 (5′–TTCGAGTTCTTTCGCGCTC–3′) as external primers and EBITS1 (5′–GCTCTGAATATCTATGGCT–3′) and EBITS2.4 (5′–ATCGCCGACGGATCCAAGTG–3′) as internal primers. TransStart^®^ Taq DNA Polymerase (TransGen Biotech, Beijing, China) was used for PCR amplifications. The cycling conditions for PCRs were: 94 °C for 5 min; followed by 35 cycles of 94 °C for 30 s, 57 °C (primary PCR) or 55 °C (secondary PCR) for 30 s, and 72 °C for 40 s; followed by 72 °C for 7 min. Positive and negative controls were included in each PCR analysis.

### Sequencing and phylogenetic analysis

Two-directional sequencing of positive PCR products was done by Sangon Biotech Co. Ltd., (Shanghai, China). The obtained nucleotide sequences were aligned with available sequences in GenBank, using ClustalX 2.1 (http://www.clustal.org/) [15]. Genotypes of *E. bieneusi* were determined based on ~243 bp of the ITS region, according to the established nomenclature system [22]. A neighbor-joining tree was generated using MEGA7 software (http://www.megasoftware.net/) [14]. The evolutionary distances were computed using the maximum composite likelihood method, and the reliability of branches in the tree was assessed by bootstrap analysis using 1000 replicates.

### Statistical analysis

Chi-square analysis was performed to assess the correlation between the prevalence of *E. bieneusi* and the age, sex, and region of pet fancy rats and guinea pigs using SPSS, version 17.0 (Statistical Package for the Social Sciences).

### Nucleotide sequence accession numbers

Unique ITS nucleotide sequences of *E. bieneusi* obtained from pet fancy rats and guinea pigs in this study were deposited in the GenBank database under accession numbers MN550998–MN551001 and MN998614–MN998615, respectively.

## Results and discussion

In the present study, *E*. *bieneusi* was detected by PCR in 17 (11.2%) of 152 pet fancy rats and 35 (20.2%) of 173 pet guinea pigs. To our knowledge, this is the first report of *E. bieneusi* infection in pet rats and pet guinea pigs worldwide. To date, there have been three studies focusing on *E. bieneusi* infection in wild rats in Iran and China [24, 30, 34] (Table 3). In this study, the prevalence of *E. bieneusi* in pet fancy rats was slightly higher than that (4.0%–8.9%) in wild rats in the above-mentioned reports. The prevalence of *E. bieneusi* in pet guinea pigs in this study was higher than that (14.9%, 10/67) in household guinea pigs in Peru [3], and also higher than other pet rodents, such as pet chinchillas (3.6%), pet squirrels (16.7%) and chipmunks (17.6%) [7, 8, 21].

**Table 3:** Prevalence and genotypes of *Enterocytozoon bieneusi* in rats (*Rattus* spp.) and guinea pigs worldwide.

Country	Host	No. examined	No. positive	Prevalence (%)	Genotypes (no.)	Reference
Iran	Wild rats (*R. norvegicus*)	146	13	8.9	D* (11), M* (2)	[24]
	Wild rats (*R. rattus*)	14	1	7.1	D (1)	
China	Wild rats (*R. norvegicus*)	242	19	7.9	D (17), Peru6* (2)	[34]
China	Wild rats (*R. norvegicus*)	199	8	4.0	CD6 (3)^a^, BEB6* (2), D (2), CHG2 (1)	[30]
China	Pet rats (*R. norvegicus*)	152	17	11.2	D (12), Peru11* (3), S7*(1), SCC-2 (1)	This study
Peru	Household guinea pigs	67	10	14.9	Peru16* (10)	[3]
China	Pet guinea pigs (*Cavia porcellus*)	173	35	20.2	S7 (30), novel genotype PGP (5)	This study

a Genotype CD6 is a synonym of genotype CHG14, and genotype S7 is a synonym of genotype CHY1, based on the nomenclature system established by Santin and Fayer [22].

* Known zoonotic genotypes: D, M, Peru6, BEB6, Peru11, Peru16, and S7.

In both pet fancy rats and guinea pigs, although the prevalences of *E. bieneusi* in younger animals and those from Weifang, Shandong were higher than those in older animals and animals from Luoyang, Henan (Tables 1 and 2), the differences in prevalence in both species between different regions, ages and sex groups were not significant (*p* > 0.05). This finding was consistent with the observations reported in a previous study on pet red-bellied tree squirrels in China [7].

In the 17 *E. bieneusi* ITS-positive samples from pet fancy rats in this study, four known genotypes were identified; genotype D (*n* = 12) was the dominant genotype, followed by Peru11 (*n* = 3), S7 (*n* = 1), and SCC-2 (*n* = 1) (Table 1). For pet guinea pigs, two genotypes were identified, including the predominant genotype S7 (*n* = 30, 85.7%) and a novel genotype (named PGP, *n* = 5) (Table 2). Until now, molecular studies of *E. bieneusi* in rats have been limited to three reports in wild rats (*R. norvegicus* and *R. rattus*) in Iran and China, and a total of six genotypes were identified, including genotypes D, CD6 (synonyms: CHG14), Peru6, M, BEB6, and CHG2 [24, 30, 34] (Table 3). For guinea pigs, only one survey conducted in Peru identified genotype peru16 from household guinea pigs (Table 3) [3], and this genotype was not detected in the present study.

In this study, genotype D was the most prevalent genotype in pet fancy rats, which is consistent with two previous reports (85.7% and 89.5%) from wild rats [24, 34], as well as other rodents such as pet red-bellied tree squirrels (*Callosciurus erythraeus*) (75.0%), pet red squirrels (*Sciurus vulgaris*) (44.3%), and domestic bamboo rats (*Rhizomys sinensis*) (77.3%) [6, 7, 26]. Genotype D is considered an important zoonotic genotype worldwide [17]. In China, genotype D has been identified in immunocompromized patients and in children with diarrhea [18, 27–29, 32]. Genotype D has also been identified in a wide range of animal hosts in China, including non-human primates, rodents (mice, rats, squirrels, chipmunks, chinchillas, and bamboo rats), other mammals (pigs, cattle, sheep, goats, alpacas, horses, donkeys, rabbits, dogs, cats, foxes, deer, takins, minks, raccoon dogs, raccoons, lions, and hippos), and birds, as well as in water samples [6, 8, 9, 11, 17, 24, 26, 30, 31, 33, 34].

In the present study, genotype Peru11, a zoonotic genotype, was identified in pet fancy rats for the first time. This genotype has been found previously in humans in Peru, China and Thailand, non-human primates in Kenya and China, raccoons, voles and cottontails in the United States, chickens in Brazil, cats in Spain, and minks and water in China [4, 5, 17, 33]. Genotype SCC-2 was reported previously in pet chipmunks and squirrels in China [6, 8], and was found in pet fancy rats for the first time. Genotype S7 (synonyms: CHY1) was originally reported in a patient in the Netherlands [25], and recently identified in a yak and pet chipmunks in China [8, 16]. This genotype was identified in a fancy rat; moreover, it was predominant in pet guinea pigs in this study, suggesting that guinea pig might be an important reservoir host of genotype S7. More studies are needed to understand the host range and public health importance of genotypes S7 and SCC-2.

**Figure 1:**
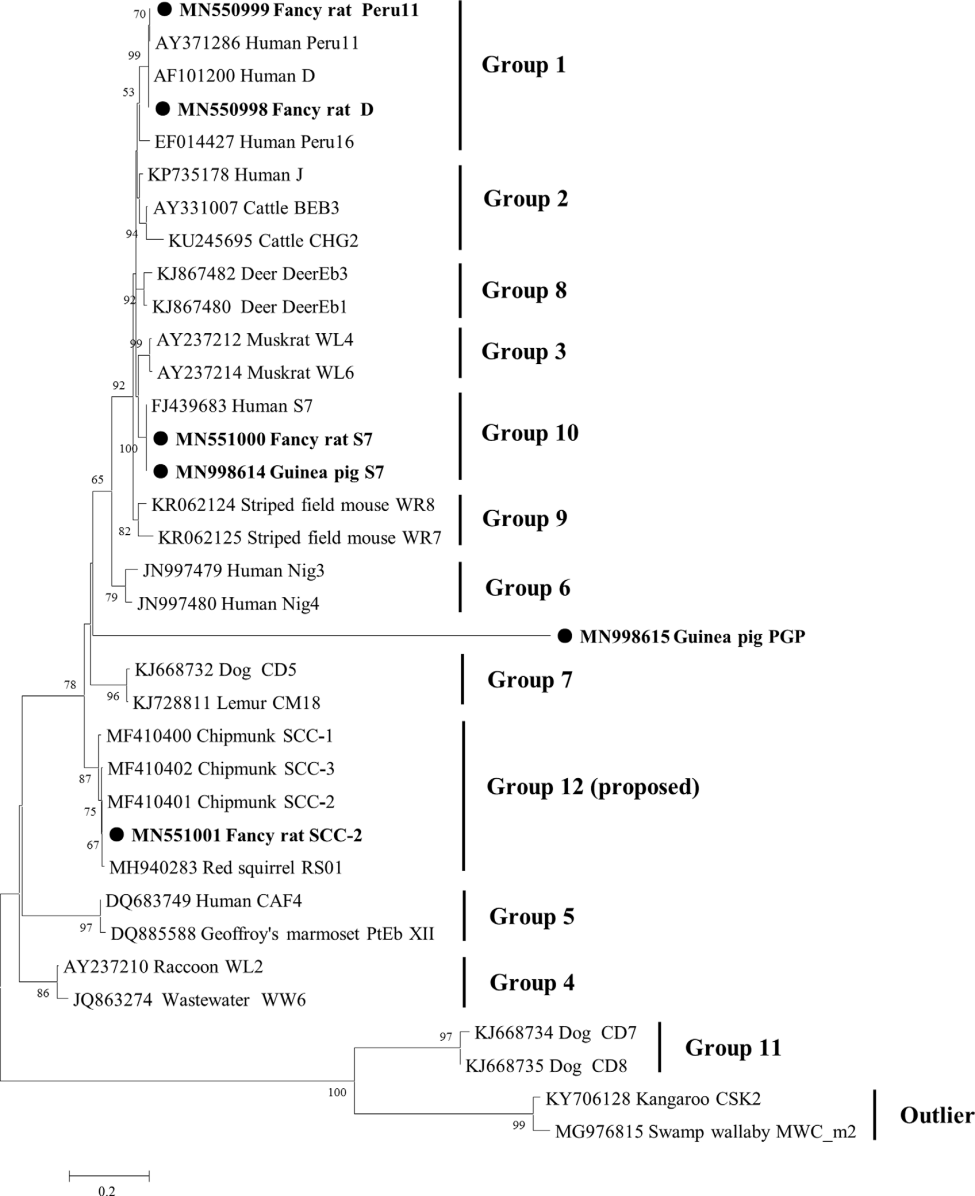
Phylogenetic relationships among the genotypes of *E. bieneusi* identified in this study and other known genotypes, as inferred by a neighbor-joining analysis of the ITS region. Bootstrap values greater than 50% from 1000 pseudoreplicates are shown. The genotypes identified in this study are indicated by closed circles.

In the phylogenetic tree of the *E. bieneusi* ITS region (Fig. 1), genotypes D and Peru11 were clustered into group 1 with strong zoonotic potential [17], and genotype S7 was clustered into group 10. Genotype SCC-2 belonged to a group which includes several chipmunk and squirrel-derived genotypes such as SCC-1–3 and RS01. Sequence and phylogenetic analysis showed that the novel genotype PGP was distinctly different. Genotype PGP exhibited less than 50% sequence similarity to the reference sequences from the known *E. bieneusi* genotype groups and outliers, i.e., 45.5% similarity to genotype CM18 in group 7, and less than 30% as compared with those in group 11 and outliers. The novel genotype PGP identified in pet guinea pigs did not cluster with any of the known *E. bieneusi* genotype groups, and formed a unique branch which was located at an intermediate position between groups 6 and 7 (Fig. 1). These data suggest that the genetic variability of *E. bieneusi* is broad, and indicate the presence of a new *E. bieneusi* genotype group; similar observations have been reported in previous studies [1, 13, 19]. Further studies on more samples collected from different regions should be conducted to understand the genetic diversity of *E. bieneusi* from rodents in China.

## Conclusions

This is the first report of *E. bieneusi* infection in pet fancy rats and pet guinea pigs. Five genotypes (D, Peru11, S7, SCC-2, and a novel genotype PGP) were identified in this study, and genotypes D and S7 were the dominant genotypes in pet fancy rats and guinea pigs, respectively. Rats (*Rattus norvegicus*) are a new host of *E. bieneusi* genotypes Peru11, S7, and SCC-2, and guinea pigs might be an important reservoir host of genotype S7. The identification of three zoonotic genotypes (D, Peru11, and S7) suggests that pet fancy rats and guinea pigs may be the sources of *E. bieneusi* infection in humans. Therefore, pet owners, especially children, should be educated to take precautions to reduce the transmission risk.

## Competing interests

The authors declare that they have no competing interests.
